# Integration of ten years of daily weather, traffic, and air pollution data from Norway’s six largest cities

**DOI:** 10.1038/s41597-024-03583-8

**Published:** 2024-07-09

**Authors:** Cong Cao

**Affiliations:** 1 Linde Center for Science, Society, and Public Policy, Division of the Humanities and Social Sciences, California Institute of Technology, 1200 E. California Blvd, Pasadena, 91125 CA USA; 2 Department of Economics, Norwegian University of Science and Technology, Høgskoleringen 1, Trondheim, Trøndelag 7034 Norway

**Keywords:** Environmental impact, Atmospheric science, Climate change

## Abstract

This study integrates ten years of daily weather, traffic, and air pollution data across the six largest Norwegian cities, utilizing data from the Norwegian Public Roads Administration, the Norwegian Institute of Air Research, and the Norwegian Meteorological Institute. The compilation of this dataset involved detailed selection and verification of monitoring stations to ensure consistency and accuracy. Initial data collection focused on the top ten most populous cities in Norway, with the subsequent examination of traffic and air pollution monitoring sites. Weather variables were then matched to the selected sites, resulting in a comprehensive dataset from 2009 to 2018. The resulting dataset encompasses extensive information, including harmful pollutants such as Nitric oxide (NO), Nitrogen dioxide (NO_2_), Nitrogen oxides (NO_*x*_), Particulate Matter less than 2.5 micrometers in diameter (PM_2.5_), and Particulate Matter less than 10 micrometers in diameter (PM_10_). The dataset’s potential for further analysis and its utility in informing policy decisions underscore its significance. This integrated dataset is a valuable resource for researchers and policymakers alike, facilitating comprehensive studies on the intersection of weather, traffic, and air pollution in urban environments.

## Background & Summary

Integrating diverse datasets spanning daily weather, traffic, and air pollution across six major Norwegian cities presents a significant endeavor to understand the complex interactions within urban environments^1^. Urban areas face increasing challenges related to climate change, air quality decline, and traffic congestion, necessitating comprehensive data analysis for informed decision-making and policy formulation^2^.

Intense traffic congestion exacerbates air quality issues by increasing traffic volume^3,4^. Air quality hazards pose significant challenges for urban residents and city administrations alike^5,6^. Research has shown that air pollution, including nitrogen oxide (*NO*_*x*_) and particulate matter, significantly contributes to mortality rates among urban populations^7,8^. These emissions primarily originate from transportation sources^9^, making the transportation sector crucial in mitigating air pollution^10^. Additionally, meteorological factors such as temperature, precipitation, wind speed, relative humidity, and atmospheric pressure significantly influence the dispersion and accumulation of particulate matter less than 2.5 micrometers in diameter(PM_2.5_)^6^. Therefore, research on the interplay between air pollution and meteorological factors affecting chronic respiratory diseases presents particular challenges in Northern Europe. As one of the Nordic countries, Norway is narrow and vast due to its geographical characteristics. Each city is located in different geographical locations, thus traffic flow and weather conditions vary significantly^11,12^. Tromsø in the north is situated within the Arctic Circle, experiencing a cold climate with reduced sunshine. Fredrikstad in the southeast is influenced by the North Sea climate, receiving more rainfall throughout the year and being prone to storms. Bergen in the west maintains high humidity year-round and is frequently battered by Atlantic storms. The capital, Oslo, located in the south, enjoys four distinct seasons and a relatively mild climate. Trondheim in the center is inland and has a colder climate. Stavanger in the southwest is influenced by the North Atlantic Warm Current, resulting in relatively warm winters but precipitation throughout the year. The differing traffic and weather conditions in these cities illustrate Norway’s diverse geography and its profound impact on residents’ lives.

This study compiles data from the Norwegian Public Roads Administration, the Norwegian Institute of Air Research, and the Norwegian Meteorological Institute, supplying a comprehensive dataset covering a decade of observations from 2009 to 2018. The dataset encompasses various pollutants such as Nitric oxide (NO), Nitrogen dioxide (NO_2_), NO_*x*_, PM_2.5_, and Particulate Matter less than 10 micrometers in diameter (PM_10_), along with detailed weather and traffic information. Through meticulous site selection and verification, a total of 18 monitoring stations were identified across the cities of Oslo, Bergen, Trondheim, Fredrikstad, Stavanger, and Tromsø.

The broader goal motivating the creation of this dataset is to facilitate multidisciplinary research endeavors and inform evidence-based policymaking. By synthesizing data from multiple sources, this study aims to clarify the complex interplay between weather patterns, traffic dynamics, and air quality in urban settings. The potential reuse value of this dataset is substantial., offering researchers and policymakers a comprehensive resource to investigate environmental trends, assess the efficacy of mitigation strategies, and develop sustainable urban planning initiatives.

This dataset holds promise for a wide range of applications, including epidemiological studies linking environmental exposures to health outcomes, transportation planning to alleviate traffic congestion, and climate modeling to assess the impact of urbanization on local weather patterns^13^. Since this dataset consists of daily data, previous studies have shown that exposure to high concentrations of NO_*x*_ and PM_2.5_, increases the incidence of cardiovascular and respiratory diseases, with a lagged effect based on the day^14^. Similar studies include Chossière *et al*.^15^, who found that high NO_*x*_ concentrations contribute to mortality^15^, and Feng *et al*.^16^, who pointed out that even lower concentrations of PM_2.5_ pose a risk to public health^16^. Therefore, the dataset is of great value in exploring the health consequences caused by traffic pollution. Overall, the integration of these datasets represents a crucial step towards enhancing our understanding of urban environmental dynamics and advancing strategies for fostering healthier, more sustainable cities^17^.

## Methods

The methodology involves several steps to generate and compile the dataset, including experimental design, data collection, analysis, and computational processing. Traffic flow, air pollution, and weather data are sourced from different providers. Compiling the dataset involved meticulous verification of geographical locations to identify common monitoring stations across the three types of data. Initially, the ten largest cities in Norway were selected based on population. Weather, pollution, and traffic data were collected from local monitoring stations for each city, prioritizing traffic and air pollution monitoring stations.

The geographical coordinates of traffic and air pollution monitoring sites were cross-validated to identify the closest matching sites, ensuring accuracy. This process was iteratively repeated for each city, resulting in the selection of monitoring stations such as Kirkeveien, Smestad, E6 Manglerud, and Alnabru in Oslo.

**Fig. 1:**
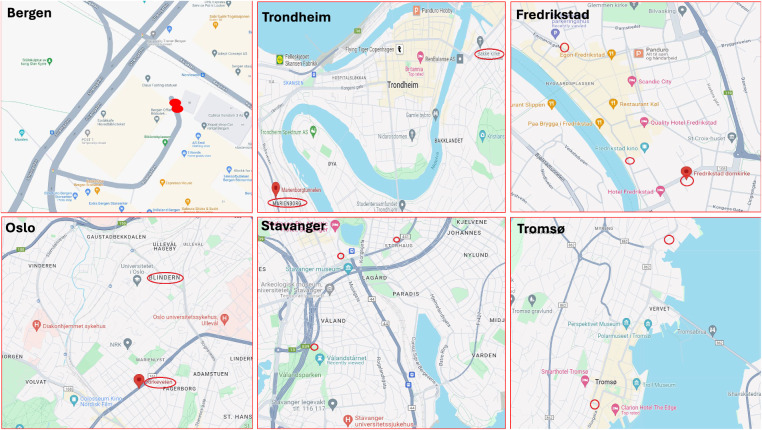
Map showing the locations of monitoring stations, sourced from a Google map, Map data ©2024 Google.

**Fig. 2:**
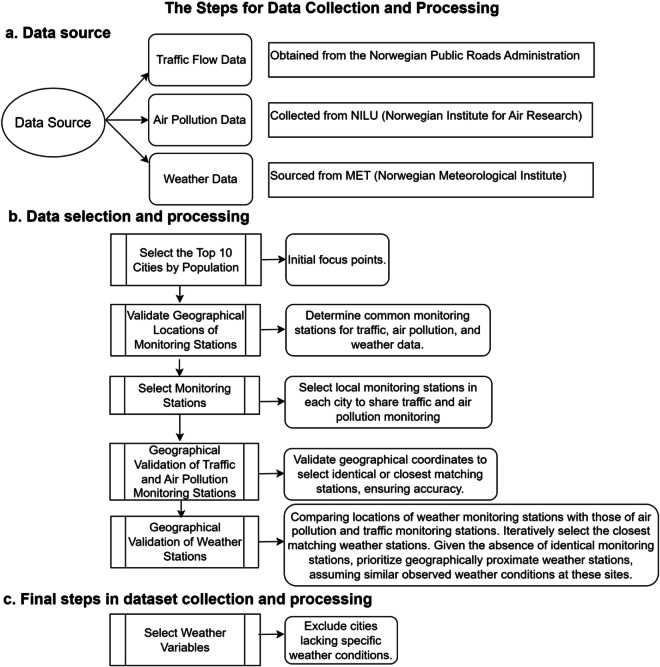
The steps for data collection and processing.

Weather variable selection involved excluding cities lacking certain weather conditions. The locations of weather monitoring stations were compared with air pollution and traffic monitoring stations to identify the nearest ones, iteratively repeating this process. Due to the absence of identical monitoring stations, especially since the distribution of weather monitoring stations differs significantly from those of the other two public data management agencies, I opted to select geographically proximate locations between weather and the other two. I assumed that the detailed weather conditions observed at these stations would be similar. Given Norway’s relatively small urban area, the meteorological conditions in areas close to traffic and pollution are also expected to be similar. Future studies could consider employing spatial network analysis for a more precise exploration, as highlighted by previous research^18,19^. For instance, Kang *et al*.^20^ utilized spatial analysis and geoAI in their study conducted in Sweden^20^. This approach enhances the capability to examine spatial relationships and geographic influences effectively. The paper provides specific distances to clarify the degree of proximity between these locations in Table 1 and Fig. 1. Figure 1 shows the locations of monitoring stations, and red hollow circles indicate the monitoring stations. Additionally, Fig. 2 outlines the steps in data collection and processing, offering a clearer and more intuitive understanding.

**Table 1:** List of stations and their distances.

City	Station Information	Walking distance/distance
Oslo	Traffic, air pollution monitor station: Oslo Kirkeveien, and weather monitoring station: Oslo Blindern	19 mins / 1.5km
Bergen	Weather and air pollution monitoring station: Danmarks. Traffic station:AMALIE/SANDVIKEN	20 mins /–
Trondheim	Air pollution and weather monitoring station: Bakke Kirke. The nearest traffic station: Marienborgtunnelen	–/3.2km
Tromsø	Air pollution and traffic data monitoring station: Hansjordnesbukta. Weather station: Domkirke	19 mins/–
Stavanger	Air pollution data monitoring station: Kannik/Kiellandsmyra. Weather station: Vålandstårnet: 13 mins walk to Kannik. Traffic station Bergelandstunnelen: 22 mins walk to Kannik.	13 mins / 12 mins
Fredrikstad	Air pollution data monitoring station: Nygaardsgata. Fredrikstad Traffic station St. Hansfjellet, 8 mins walk to Nygaardsgata. Fredrikstad weather station: Fredrikstad Domkirke, 3 mins walk to Nygaardsgata.	8 mins / 3 mins

The selected analysis period covers daily data from 2009 to 2018. The dataset includes maximum and minimum normalized data after data imputation using the R package “mice”, ensuring no missing values. Data from Oslo, Bergen, and Stavanger spanning 2007 to 2018 were collected using the same methodology for subsequent analysis.

Given that data collection occurred just as COVID-19 was concluding or nearing its conclusion, it’s crucial to acknowledge that traffic patterns and associated air pollution likely fluctuated during the pandemic. Therefore, data from the period before COVID-19 was selected for analysis. Additionally, annual air pollution data undergoes quality assurance procedures before July 1 of the following year; before this date, adjustments may occur due to ongoing quality control measures. Additionally, hourly data from Oslo’s Bygdøy for the first half of 2019 was collected, with missing data on May 17th supplemented.

**Fig. 3:**
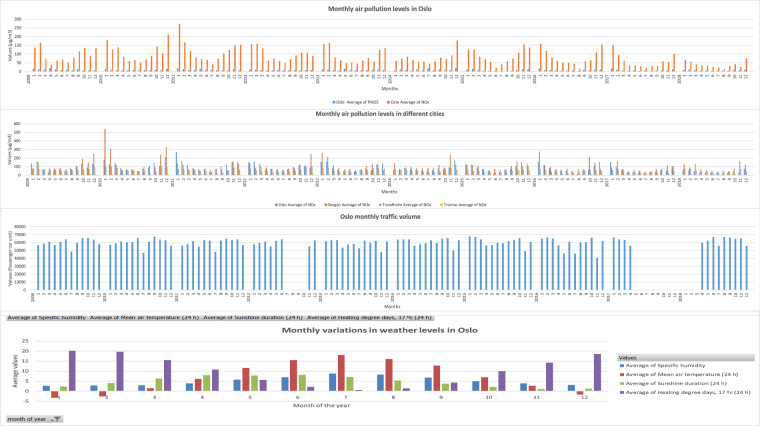
Visualization of aggregated monthly data for Oslo’s traffic and air pollution, along with NOx values from selected cities.

To avoid overcrowding the horizontal axis with daily data points, the data was initially aggregated into monthly intervals. Subsequently, selected data points were sampled for visualization. Figure 3 presents visualizations for traffic volume (TV) and air pollution data in Oslo, as well as NO_*x*_ values from several other chosen cities.

## Data Records

The data presented in the repository consists of comprehensive information on daily weather, traffic, and air pollution across six major Norwegian cities over ten years, from 2009 to 2018. The dataset was compiled from multiple sources, including the Norwegian Public Roads Administration, the Norwegian Institute of Air Research, and the Norwegian Meteorological Institute.

The dataset is available at the Scientific Data Bank platform at the following link: https://www.scidb.cn/en/detail?dataSetId=5eb39369148d4f40bb5ec398d6503c46^21^. The data is provided in Excel or CSV format, ensuring compatibility with various analysis tools and software packages. Here’s a summary of the data records:

1. Data Content: The dataset includes information on common pollutants such as NO_*x*_, NO, PM_2.5_, etc, along with detailed weather variables including air temperature, dew point temperature, cloud cover, wind speed, humidity, precipitation, and snow cover.
2. Repository Structure: The repository contains separate files for each city, with data organized in a tabular format. Each file corresponds to a specific monitoring station within the respective city.
3. File Naming Convention: The files are named according to the city and the type of data source. For example, “Oslo_traffic.csv” contains traffic data from Oslo.
4. Data Availability: The dataset covers daily observations for each variable from 2009 to 2018.
5. Data Validation: The dataset underwent thorough validation to ensure accuracy and consistency. This involved meticulous site selection, verification of geographical locations, and comparison of monitoring stations across different data sources. Missing values were imputed using the R package “mice”.
6. Code Availability: The code used for data analysis and processing is available on the author’s GitHub repository at the following link: https://github.com/congca/Scientific-data-publishand 10.5281/zenodo.11649590. Readers can navigate the repository to access specific city data files, explore the variables of interest, and utilize the dataset for various research purposes, including environmental studies, urban planning, and policy formulation.

**Fig. 4:**
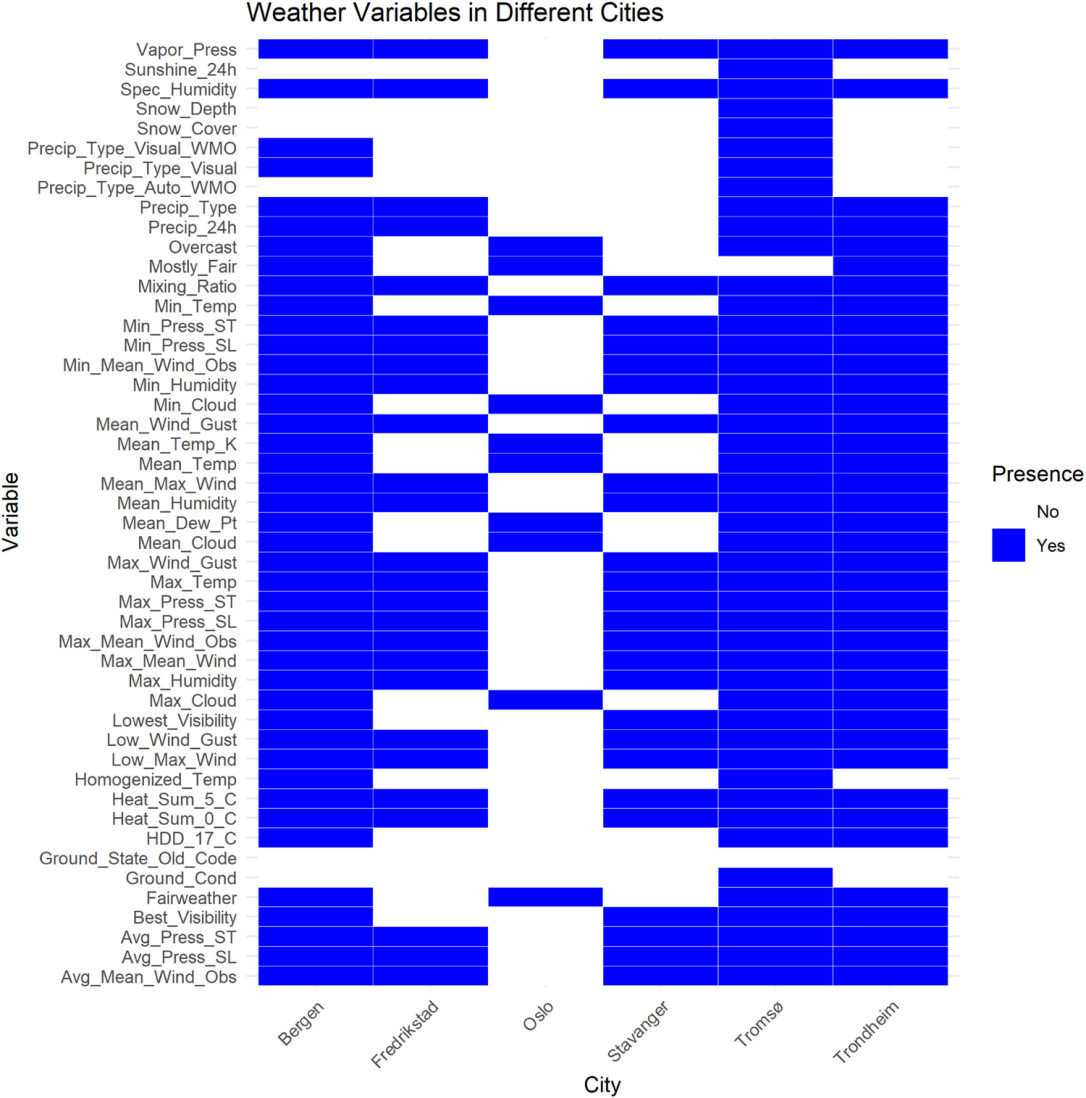
Weather variables in different cities.

Tables 2 to 3, and Appendix 1 provide comprehensive descriptions of the variables (columns) included in the traffic, air pollution, and weather datasets. These tables offer detailed information about each variable, including their names, units of measurement, and the specific data they represent. In Fig. 4 heatmap, the color blue denotes the presence of variables, whereas white indicates their absence. This visualization offers a clear representation of the occurrence of different weather variables across each city. They clarify the abbreviations used in the datasets and enumerate the specific weather-related variables available for each city. It is important to note that all cities include data variables related to both traffic and air pollution, ensuring a consistent dataset for comparative analysis and research across different urban areas. Table 4 presents a detailed breakdown of urban and countryside locations within various cities. This table categorizes specific monitoring sites and their corresponding environmental contexts, providing valuable insights into the spatial distribution of data collection points. By distinguishing between urban and countryside locations, this table facilitates a deeper understanding of how geographic context influences traffic and air pollution data. The variable abbreviations are provided in Table 5.

**Table 2:** Variable descriptions for “Traffic.csv” files.

Variable	Description
Trafikkregistreringspunkt (Traffic registration point)	Unique ID of the point.
Navn (Name)	Name of the monitoring point.
Vegreferanse (Road reference)	Location of the point on the road network.
Fra (From)	Time of start of monitoring.
Til (To)	Time of end of monitoring and data collection.
Dato (Date)	Date of monitoring.
Felt (Field)	The lane of travel in which the vehicle was registered represents the actual position on the road.
Volum (Volume)	Average volume of traffic per day (number of vehicles).
Dekningsgrad (%) (Degree of coverage (%))	Proportion of the time interval for which traffic registrations have data, accounting for measurement uncertainty.
Antall timer total (Number of hours total)	Total number of hours or days in the period.
Antall timer inkludert (Number of hours included)	Number of hours for which data is available during the period.
Antall timer ugyldig (Number of hours invalid)	Number of hours in the time period for which the quality is unknown.
Ikke gyldig lengde (Not valid length)	Number of vehicles that were assessed as having failed length measurements and are not included in the length classification.
Lengdekvalitetsgrad (%) (Length quality degree (%))	Percentage of vehicles that have approved length measurements.
< 5.6m	Number of vehicles with a measured length less than 5.6 m.
>= 5.6m	Number of vehicles with a measured length greater than or equal to 5.6 m.
5.6m - 7.6m	Number of vehicles with a measured length between 5.6 m and 7.6 m.
7.6m - 12.5m	Number of vehicles with a measured length between 7.6 m and 12.5 m.
12.5m - 16.0m	Number of vehicles with a measured length between 12.5 m and 16.0 m.
16.0m - 24.0m	Number of vehicles with a measured length between 16.0 m and 24.0 m.
> 24.0m	Number of vehicles with a measured length greater than or equal to 24.0 m (valid up to 27.0 m).

**Table 3:** Description of variables in the “pollution.csv” file.

Variable	Description
Tid	Time
Danmarks plass NO_*x*_ *μ*g/m^3^ dag	Danmarks plass NOx *μ*g/m^3^ Day; NOx concentration measured in *μ*g/m^3^ at Danmarks plass monitoring site, daily values
Dekning	Coverage; constant value of 100
Danmarks plass PM_2.5_ *μ*g/m^3^ dag	Danmarks plass PM_2.5_ *μ*g/m^3^ Day; PM_2.5_ concentration measured in *μ*g/m^3^ at Danmarks plass monitoring site, daily values

**Table 4:** The urban and countryside locations in various cities.

Kirkeveien	
Smestad	countryside
E6 Manglerud	countryside
Alnabru	countryside

Bergen	
1. Danmarks Plass	city
2. Loddefjord	countryside
3. Rådal	countryside
4. Åsane	city

Stavanger	
1. Kannik	countryside
2. Schancheholen	Regional

Trondheim	
1. Bakke Kirke	city
2. E6 Tiller ved XXL	countryside
3. Elgeseter	city

Fredrikstad	
St. Croix	countryside

**Table 5:** Abbreviations and Full Terms.

Abbreviation	Full Term
Fairweather	Fairweather (24 h)
Mostly Fair	Mostly fair weather (24 h)
Overcast	Overcast weather (24 h)
Max Cloud	Maximum Cloud Cover (24 h)
Mean Cloud	Mean cloud cover (24 h)
Min Cloud	Minimum cloud cover (24 h)
Min Temp	Minimum air temperature (24 h)
Mean Temp (K)	Mean air temperature, Köppen’s formula (24 h)
Mean Dew Pt	Mean dew point temperature (24 h)
Mean Temp	Mean air temperature (24 h)
Homogenized Temp	Homogenised mean temperature (24 h)
HDD (17 ^∘^C)	Heating degree days, 17 ^∘^C (24 h)
Heat Sum (0 ^∘^C)	Heat sum, 0 ^∘^C (døgn)
Heat Sum (5 ^∘^C)	Heat sum, 5 ^∘^C (døgn)
Max Temp	Maximum air temperature (24 h)
Max Press (SL)	Maximum air pressure, sea level (24 h)
Max Press (ST)	Maximum air pressure, station level (24 h)
Avg Press (SL)	Average air pressure, sea level (24 h)
Avg Press (ST)	Average air pressure, station level (24 h)
Vapor Press	Vapour pressure (24 h)
Min Press (SL)	Minimum air pressure, sea level (24 h)
Min Press (ST)	Minimum air pressure, station level (24 h)
Max Mean Wind	Maximum mean wind speed (24 h)
Max Mean Wind (Obs)	Maximum mean wind speed from main obs. (24 h)
Max Wind Gust	Maximum wind gust (24 h)
Mean Max Wind	Mean of maximum mean wind speed (24 h)
Avg Mean Wind (Obs)	Average of mean wind speed from main obs. (24 h)
Mean Wind Gust	Mean wind gust (24 h)
Low Max Wind	Lowest maximum mean wind speed (24 h)
Min Mean Wind (Obs)	Minimum mean wind speed from main obs. (24 h)
Low Wind Gust	Lowest wind gust (24 h)
Max Humidity	Maximum relative humidity (24 h)
Mean Humidity	Mean relative humidity (24 h)
Min Humidity	Minimum relative humidity (24 h)
Mixing Ratio	Mixing ratio
Spec Humidity	Specific humidity
Best Visibility	Best visibility (24 h)
Lowest Visibility	Lowest visibility (24 h)
Snow Cover	Snow cover
Snow Depth	Snow depth
Precip Type (Visual)	Precipitation type visual
Precip Type	Precipitation type
Precip Type (Auto WMO)	Precipitation type automatic WMO
Precip Type (Visual WMO)	Precipitation type visual WMO
Precip (24 h)	Precipitation (24 h)
Ground State (Old Code)	State of ground, older international code standard
Ground Cond.	Ground condition
Sunshine (24 h)	Sunshine duration (24 h)

## Technical Validation

Compiling the final dataset proved time-consuming due to the disparate sources of traffic, pollution, and weather data. The objective was to identify common monitoring stations or, at least, those near each other across the three types of data sources. As a result, the process involved meticulous verification and comparison of geographical locations using Google Maps.

Initially, data collection began by selecting the ten largest cities in Norway based on population. Subsequently, data from local weather, pollution, and traffic monitoring stations were gathered from the respective government websites for each city. Each city boasted numerous monitoring stations for each type of data. The process prioritized traffic and air pollution monitoring stations.

To ensure accuracy, the geographical coordinates of traffic and air pollution monitoring sites were cross-referenced and verified against each other, thereby identifying the closest matching sites. This iterative process was repeated for every city, culminating in the selection of monitoring sites such as Kirkeveien, Smestad, E6 Manglerud, and Alnabru in Oslo; Danmarks Plass, Loddefjord, Rådal, and Åsane in Bergen; Kannik and Schancheholen in Stavanger; and Bakke Kirke, E6 Tiller ved XXL, and Elgeseter in Trondheim.

Additionally, the air pollutant monitoring stations adjacent to the highway were chosen to exclusively study traffic-related air pollution, mitigating other potential sources of pollution. This meticulous site selection process resulted in a total of 18 monitoring stations across various cities, with the earliest available data dating back to February 1, 1996, provided in hourly intervals. The common pollutants monitored across these sites include NO, NO_2_, NO_*x*_, PM_2.5_, and PM_10_, underscoring the significance of the selected monitoring sites and the effort invested in their identification. The information provided here serves as a testament to the value derived from the time-intensive process of identifying these monitoring sites, updated as of July 20, 2022.

Following that, the selection process turned to weather variables. However, the weather data collection website operates with a system wherein variables are chosen before selecting the city, resulting in a reversed order of selection. Given that there are more weather variables than pollutants and traffic, if all the stations in a particular city lack certain weather conditions, that city is excluded. Consequently, only six cities remained in the dataset. Stavanger was initially included but was subsequently removed due to a significant proportion of missing values in certain weather variables upon download.

**Fig. 5:**
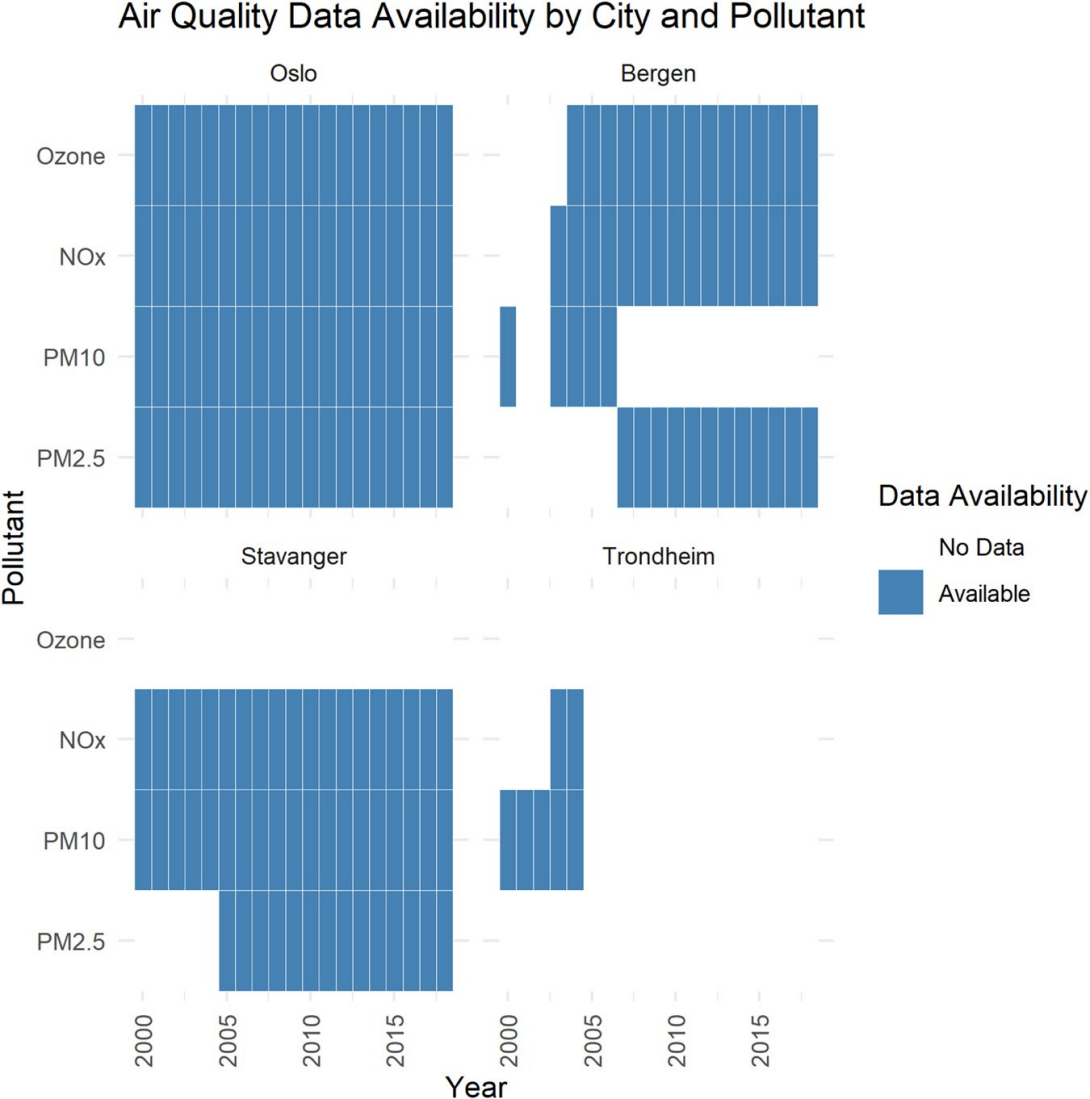
Air Quality Data Availability by City and Pollutant.

The subsequent step involved comparing the locations of weather monitoring stations with air pollution and traffic monitoring stations to identify the nearest one. This process was repeated iteratively. The next consideration was to check the time to find the highest frequency but longest period available. The air quality data availability by city and pollutant is in Fig. 5.

The period chosen is from 2009 to 2018, with daily data. All data is stored in Excel or CSV format and can be found at Scientific data bank. Tables in the data records section provide essential details about the main data inputs and variables. The data underwent maximum and minimum normalization, followed by data imputation using the R package “mice” to ensure no missing values. Additionally, data was collected from Oslo, Bergen, and Stavanger spanning 2007 to 2018 using the same methodology for further analysis. Monthly plot covering traffic volume (TV), NO_*x*_, and PM_2.5_ readings from Oslo, Bergen, Trondheim, and Tromsø over ten years is also available.

### Two standard check

Studies have utilized this dataset for related research. For instance, Cao (2024) examined hourly data from the same source to investigate interactions among air pollution, traffic, and weather, comparing machine learning algorithms with traditional methods^1^. Furthermore, Cao *et al*. (2024) utilized a subset of this dataset to analyze key meteorological and traffic factors influencing air pollutants, assessing the predictive accuracy of physics-based deep learning and long short-term memory (LSTM)^17^.

All the data sources are freely available. Air pollution data is obtained from https://luftkvalitet.nilu.no/en/historical. The website offers historical air quality data verified by municipal authorities and the National Air Quality Reference Laboratory, including preliminary data for the current year. You can view charts or download data in CSV format, select specific dates, times, stations, or components, and choose from various averages. Data can be accessed at http://api.nilu.no for personal use and should be properly referenced in your work. When referencing NILU air quality data for Oslo in 2023, cite it as NILU - Norwegian Institute for Air Research. (2023). Air Quality Data for Oslo, January 2023 - December 2023. Available at: http://api.nilu.no.

Weather data is sourced from: https://seklima.met.no/observations/. When using meteorological data provided by the Norwegian Climate Service Center (https://klimaservicesenter.no/), please ensure that you accurately cite them as the source in your research or course materials. This includes listing the Norwegian Climate Service Center in your references and providing a link to their website, allowing readers or viewers to verify the data source.

Traffic flow data is obtained from the Norwegian Public Roads Administration’s website, specifically from https://trafikkdata.atlas.vegvesen.no/#/kart?lat=62.59346885386541&lon=10.86809745774079&trafficType=vehicle&zoom=4.

Use of the traffic data API is governed by the Norwegian Open Government Data License (NLOD), allowing copying, distribution, modification, and commercial use, with considerations for data quality, potential errors, and omissions. The Norwegian Public Roads Administration does not guarantee data accuracy or relevance and assumes no liability for any losses from its use.

### Supplementary information

Variables descriptions for "weather.csv" files

## Data Availability

The dataset is available from: https://www.scidb.cn/en/detail?dataSetId=5eb39369148d4f40bb5ec398d6503c46 or 10.57760/sciencedb.17721. The code to reproduce the analysis is available at Github repository with 10.5281/zenodo.11649590.
