# Supplementary material for: Integration of ten years of daily weather, traffic, and air pollution data from Norway’s six largest cities
Source: Sci Data. 2024 Jul 9;11:744. doi: 10.1038/s41597-024-03583-8 (PMC11233595; doi:10.1038/s41597-024-03583-8)
Supplement: Supplementary file 1 — Variables descriptions for "weather.csv" files [file 41597_2024_3583_MOESM1_ESM.pdf]

Appendix 1

Table 1. Variables descriptions for weather.csv files

Name	Description
Station Time (Norwegian meantime)	The name of the weather monitoring station
Homogenised mean temperature °C (24 h)	Daily average homogeneous temperature, the average value is the arithmetic mean of 24 hours
Heating degree days, 17 ºC (24 h)	This value indicates how much the hourly average temperature is below 17 degrees; if it is equal to or greater than 17 degrees, the value is 0; it also indicates the energy demand for indoor heating
Heat sum, 0 ºC (døgn)	Daily heat sum, also known as growing degree day, is a measure of plant development conditions, where the base temperature is 0 degrees, indicating the degree above the base temperature
Heat sum, 5 ºC (døgn)	Daily heat sum, where the base temperature is 5 degrees, indicating the degree above the base temperature
Maximum air temperature °C (24 h)	Highest temperature per 24 hours
Mean air temperature °C (24 h)	The average of ambient air temperature 2 meters above the ground and the current value
Mean dew point temperature °C (24 h)	The temperature to which air needs to be cooled at a constant pressure to achieve a relative humidity of 100%
Mean air temperature, Köppen's formula ºC(24 h)	Calculation of monthly mean temperature by Köppen's formula in the Norwegian station network [**?**]
Minimum air temperature °C (24 h)	Lowest temperature per 24 hours
Fairweather (24 h)	Fairweather, binary categorical variable, 0 or 1; if cloud cover is not greater than 4, it is sunny, the parameter value is set to 1, otherwise it is 0
Mostly fair weather (24 h)	Partly fair weather, binary categorical variable, 0 or 1; if the cloud cover is greater than 5 and less than 9, it is partly clear, the parameter value is set to 1, otherwise it is 0
Maximum cloud cover (24 h)	The highest cloud cover recorded every 24 hours, cloud cover value is 20 or larger
Overcast weather (24 h)	Overcast weather; if the cloud cover is not less than 20, it is overcast, the parameter value is set to 1, otherwise it is 0
Mean cloud cover (24 h)	Daily average cloud cover, the arithmetic mean of the cloud cover at 6, 12, and 18 o'clock
Minimum cloud cover (24 h)	The minimum cloud cover recorded every 24 hours
Maximum air pressure, sea level kPa (24 h)	Air pressure reduced to sea level, daily maximum value; measured air pressure reduced to mean sea level by applying actual atmospheric conditions
Maximum air pressure, station level kPa (24 h)	Air pressure at the monitoring station, daily maximum value, measured air pressure reduced to the measured air pressure at the station
Average air pressure, sea level kPa (24 h)	The air pressure is obtained by reducing the air pressure at the sea level
Average air pressure, station level kPa (24 h)	The air pressure obtained by reducing the air pressure at the measuring station
Vapour pressure kPa (24 h)	Daily average vapor pressure obtained based on relative humidity and current temperature
Minimum air pressure, sea level kPa (24 h)	Air pressure converted to sea level, minimum value every 24 hours
Minimum air pressure, station level kPa (24 h)	The minimum value of air pressure at the monitoring station every 24 hours
Maximum mean wind speed m/s (24 h)	Daily maximum mean wind speed
Maximum mean wind speed from main obs. m/s (24 h)	Daily maximum mean wind speed observed every hour
Maximum wind gust m/s (24 h)	Daily maximum gust

Name	Description
Mean of maximum mean wind speed m/s (24 h)	Daily mean of hourly maximum mean wind speed
Average of mean wind speed from main obs. m/s (24 h)	Average wind speed of main observation points
Mean wind gust m/s (24 h)	Daily average gust
Lowest maximum mean wind speed m/s (24 h)	Daily minimum of the hourly maximum mean wind speed
Minimum mean wind speed from main obs. m/s (24 h)	Daily minimum mean wind speed of hourly observations
Lowest wind gust m/s (24 h)	Daily minimum gust
Maximum relative humidity (24 h)	Maximum relative humidity per 24 hours
Mean relative humidity % (24 h)	Daily mean relative humidity
Minimum relative humidity %(24 h)	Daily minimum relative humidity
Mixing ratio (%)	The ratio of the absolute humidity in the air to the saturated absolute humidity at the same temperature and pressure
Specific humidity	The amount of water vapor present in the air
Best visibility (24 h)	Maximum visibility per day, converted from synop code or metar code
Lowest visibility (24 h)	Minimum visibility per day, converted from synop code or metar code
Snow cover (code 0-4)	Snow cover is registered as a code 0-4, 1 = mostly snow-free; 2 = equal amounts of snow and snow-free ground; 3 = mostly covered with snow; 4 = completely covered with snow
Snow depth (cm)	Daily total snow measurement from the ground to the top of the snow cover
Precipitation (cm)	Daily precipitation sum
Precipitation type (visual)	A visual summary of the precipitation type for the past 24 hours based on the weather elements using the weather code table
Precipitation type	A summary of the precipitation type for the past 24 hours
